# fNIRS reproducibility varies with data quality, analysis pipelines, and researcher experience

**DOI:** 10.1038/s42003-025-08412-1

**Published:** 2025-08-04

**Authors:** Meryem A. Yücel, Robert Luke, Rickson C. Mesquita, Alexander von Lühmann, David M. A. Mehler, Michael Lührs, Jessica Gemignani, Androu Abdalmalak, Franziska Albrecht, Iara de Almeida Ivo, Christina Artemenko, Kira Ashton, Paweł Augustynowicz, Aahana Bajracharya, Elise Bannier, Beatrix Barth, Laurie Bayet, Jacqueline Behrendt, Hadi Borj Khani, Lenaic Borot, Jordan A. Borrell, Sabrina Brigadoi, Kolby Brink, Chiara Bulgarelli, Emmanuel Caruyer, Hsin-Chin Chen, Christopher Copeland, Isabelle Corouge, Simone Cutini, Renata Di Lorenzo, Thomas Dresler, Adam T. Eggebrecht, Ann-Christine Ehlis, Sinem B. Erdoğan, Danielle Evenblij, Talukdar Raian Ferdous, Victoria Fracalossi, Erika Franzén, Anne Gallagher, Christian Gerloff, Judit Gervain, Noy Goldhamer, Louisa K. Gossé, Ségolène M. R. Guérin, Edgar Guevara, SM Hadi Hosseini, Hamish Innes-Brown, Isabell Int-Veen, Sagi Jaffe-Dax, Nolwenn Jégou, Hiroshi Kawaguchi, Caroline Kelsey, Michaela Kent, Roman Kessler, Nadeen Kherbawy, Franziska Klein, Nofar Kochavi, Matthew Kolisnyk, Yogev Koren, Agnes Kroczek, Alexander Kvist, Chen-Hao Paul Lin, Andreas Löw, Siying Luan, Darren Mao, Giovani G. Martins, Eike Middell, Samuel Montero-Hernandez, Murat Can Mutlu, Sergio L. Novi, Natacha Paquette, Ishara Paranawithana, Yisrael Parmet, Jonathan E. Peelle, Ke Peng, Tommy Peng, João Pereira, Paola Pinti, Luca Pollonini, Ali Rahimpour Jounghani, Vanessa Reindl, Wiebke Ringels, Betti Schopp, Alina Schulte, Martin Schulte-Rüther, Ari Segel, Tirdad Seifi Ala, Maureen J. Shader, Hadas Shavit, Arefeh Sherafati, Mojtaba Soltanlou, Bettina Sorger, Emma Speh, Kevin D. Stubbs, Katharina Stute, Eileen F. Sullivan, Sungho Tak, Zeus Tipado, Julie Tremblay, Homa Vahidi, Maaike Van Eeckhoutte, Phetsamone Vannasing, Gregoire Vergotte, Marion A. Vincent, Eileen Weiss, Dalin Yang, Gülnaz Yükselen, Dariusz Zapała, Vit Zemanek

**Affiliations:** 1 Boston University, Neurophotonics Center, Boston, MA USA; 2 Department of Biomedical Engineering, College of Engineering, Boston University, Boston, MA USA; 3 Department of Linguistics, Macquarie University, Sydney, NSW Australia; 4 Australia Hearing Hub, Macquarie University Hearing, Sydney, NSW Australia; 5 School of Computer Science, University of Birmingham, Birmingham, UK; 6 Institute of Physics, University of Campinas (UNICAMP), Campinas, São Paulo, Brazil; 7 Intelligent Biomedical Sensing (IBS) Lab, Machine Learning Department, TU Berlin, Berlin, Germany; 8 BIFOLD – Berlin Institute for Foundations of Learning and Data, Berlin, Germany; 9 Applied Computational Neuroscience (ACN) lab, Department for Psychiatry, Psychotherapy and Psychosomatics, Medical School, RWTH Aachen University, Aachen, Germany; 10 Institute of Translational Psychiatry, Medical Faculty, University of Münster, Münster, Germany; 11 School of Psychology, Cardiff University Brain Research Imaging Center (CUBRIC), Cardiff University, Cardiff, UK; 12 Department of Cognitive Neuroscience, Maastricht University, Maastricht, The Netherlands; 13 Department of Developmental Psychology and Socialization, Università degli Studi di Padova, Padua, Italy; 14 Padova Neuroscience Center, Padua, Italy; 15 Brain and Mind Institute, Western University, London, ON Canada; 16 Department of Physiology and Pharmacology, Western University, London, ON Canada; 17 Division of Physiotherapy, Department of Neurobiology, Care Sciences and Society, Karolinska Institutet, Stockholm, Sweden; 18 Karolinska University Hospital, Women’s Health and Allied Health Professionals Theme, Medical Unit Occupational Therapy, Stockholm, Sweden; 19 Department of Psychology, University of Tübingen, Tübingen, Germany; 20 Department of Neuroscience, American University, Washington, WA USA; 21 Department of Experimental Psychology, The John Paul II Catholic University of Lublin, Lublin, Poland; 22 Mallinckrodt Institute of Radiology, Washington University School of Medicine, St. Louis, WA USA; 23 CHU Rennes, Radiology Department, Rennes, France; 24 Univ Rennes, Inria, CNRS, Inserm, IRISA UMR 6074, Empenn, Rennes, France; 25 Department of Psychiatry and Psychotherapy, Tübingen Center for Mental Health, University of Tübingen, Tübingen, Germany; 26 LEAD Graduate School & Research Network, University of Tübingen, Tübingen, Germany; 27 German Center for Mental Health (DZPG), partner site, Tübingen, Germany; 28 BEARLabs, Faculty 1, University of Applied Sciences (HTW) Berlin, Berlin, Germany; 29 Research Institute for Sport and Exercise Sciences, Liverpool John Moores University, Liverpool, England; 30 Department of Biomechanics, University of Nebraska at Omaha, Omaha, NE USA; 31 Department of Occupational Therapy Education, University of Kansas Medical Center, Kansas City, KS USA; 32 Centre for Brain and Cognitive Development, Birkbeck, University of London, London, UK; 33 Department of Psychology, National Chung Cheng University, Minhsiung, Chiayi, Taiwan; 34 Division of Developmental Medicine, Department of Pediatrics, Boston Children’s Hospital, Boston, MA USA; 35 Harvard Medical School, Boston, MA USA; 36 Faculty of Engineering and Natural Sciences, Department of Biomedical Engineering, Acıbadem Mehmet Ali Aydınlar University, Istanbul, Turkey; 37 Department of Biomedical Engineering, University of Houston, Houston, TX USA; 38 Department of Psychology, University of Montreal, Montreal, QC Canada; 39 Department of Child and Adolescent Psychiatry, RWTH Aachen University, Aachen, Germany; 40 JARA-Brain Institute II, Molecular Neuroscience and Neuroimaging, RWTH Aachen & Research Centre Juelich, Aachen, Germany; 41 The Translational Neurorehabilitation Lab at Adi Negev Nahalat Eran, Ofakim, Israel; 42 Institute of Neuroscience, Université Catholique de Louvain, Brussels, Belgium; 43 Sciences Cognitives et Sciences Affectives, University of Lille, CNRS, Lille, France; 44 CIACYT, Universidad Autónoma de San Luis Potosí, San Luis Potosí, Mexico; 45 Department of Psychiatry and Behavioral Sciences, Computational Brain Research and Intervention (C-BRAIN) Lab, Stanford University, Stanford, CA USA; 46 Eriksholm Research Centre, Snekkersten, Denmark; 47 Department of Health Technology, Technical University of Denmark, Lyngby, Denmark; 48 School of Psychological Sciences and Sagol School of Neuroscience, Tel Aviv University, Tel Aviv, Israel; 49 Human Informatics and Interaction Research Institute, National Institute of Advanced Industrial Science and Technology (AIST), Tsukuba, Japan; 50 Schulich School of Medicine and Dentistry, Western University, London, ON Canada; 51 Department of Neuropsychology, Max Planck Institute for Human Cognitive and Brain Sciences, Leipzig, Germany; 52 Neurocognition and functional Neurorehabilitation Group, Department of Psychology, University of Oldenburg, Oldenburg, Germany; 53 Biomedical Devices and Systems Group, R&D Division Health, OFFIS Institute for Information Technology, Oldenburg, Germany; 54 Department of Psychology, Western University, London, ON Canada; 55 Department of Physical Therapy, Ben-Gurion University, Beersheba, Israel; 56 Department of Physics, Washington University in St. Louis, St. Louis, MO USA; 57 Experimental Psychology Unit, Helmut Schmidt University/University of the Federal Armed Forces Hamburg, Hamburg, Germany; 58 Health and Rehabilitation Sciences Program, Faculty of Health Science, Western University, London, ON Canada; 59 Human Hearing, The Bionics Institute, East Melbourne, VIC Australia; 60 Institute of Biology, Otto von Guericke University, Magdeburg, Germany; 61 Industrial engineering and management, Ben-Gurion university, Beersheba, Israel; 62 Department of Communication Sciences and Disorders and Department of Psychology, Northeastern University, Boston, MA USA; 63 Department of Electrical and Computer Engineering, Price Faculty of Engineering, University of Manitoba, Winnipeg, MB Canada; 64 Coimbra Institute for Biomedical Imaging and Translational Research, Institute for Nuclear Sciences Applied to Health – University of Coimbra, Coimbra, Portugal; 65 Department of Engineering Technology, University of Houston, Houston, TX USA; 66 Department of Psychology, University of California, Merced, Merced, CA USA; 67 Psychology, School of Social Sciences, Nanyang Technological University, Singapore, Republic of Singapore; 68 Department of Experimental Otology of the Clinics of Otolaryngology, Hannover Medical School, Hannover, Germany; 69 Department of Child and Adolescent Psychiatry and Psychotherapy, University Medical Center Göttingen, Göttingen, Germany; 70 Department of Child and Adolescent Psychiatry and Psychotherapy, University Hospital Heidelberg, Heidelberg, Germany; 71 Department of Speech, Language, and Hearing Sciences, Purdue University, West Lafayette, IN USA; 72 Department of Neurology, University of California, San Francisco, CA USA; 73 School of Psychology, University of Surrey, Guildford, UK; 74 Department of Childhood Education, Faculty of Education, University of Johannesburg, Johannesburg, South Africa; 75 BrainsCAN, Western University, London, ON Canada; 76 Chemnitz University of Technology, Faculty of Behavioural and Social Sciences, Department of Human Movement Science and Health, Chemnitz, Germany; 77 Center for Bio-Imaging and Translational Research, Korea Basic Science Institute, Cheongju, Republic of Korea; 78 Graduate School of Analytical Science and Technology, Chungnam National University, Daejeon, Republic of Korea; 79 Department of Psychopharmacology, Maastricht University, Maastricht, The Netherlands; 80 Copenhagen Hearing and Balance Centre, Rigshospitalet, Copenhagen, Denmark; 81 EuroMov Digital Health in Motion, Univ Montpellier, IMT Mines Ales, Montpellier, France; 82 Department of Clinical Neuropsychology, University Hospital RWTH Aachen, Aachen, Germany; 83 Faculty of Science, University of Amsterdam, Amsterdam, The Netherlands

**Keywords:** Neuroscience, Research data

## Abstract

As data analysis pipelines grow more complex in brain imaging research, understanding how methodological choices affect results is essential for ensuring reproducibility and transparency. This is especially relevant for functional Near-Infrared Spectroscopy (fNIRS), a rapidly growing technique for assessing brain function in naturalistic settings and across the lifespan, yet one that still lacks standardized analysis approaches. In the fNIRS Reproducibility Study Hub (FRESH) initiative, we asked 38 research teams worldwide to independently analyze the same two fNIRS datasets. Despite using different pipelines, nearly 80% of teams agreed on group-level results, particularly when hypotheses were strongly supported by literature. Teams with higher self-reported analysis confidence, which correlated with years of fNIRS experience, showed greater agreement. At the individual level, agreement was lower but improved with better data quality. The main sources of variability were related to how poor-quality data were handled, how responses were modeled, and how statistical analyses were conducted. These findings suggest that while flexible analytical tools are valuable, clearer methodological and reporting standards could greatly enhance reproducibility. By identifying key drivers of variability, this study highlights current challenges and offers direction for improving transparency and reliability in fNIRS research.

## Abstract (web summary)

FRESH, a multi-lab collaboration, reveals how data quality, analysis pipelines, and researcher expertise influence reproducibility in fNIRS neuroimaging, highlighting key factors driving variability in hypothesis outcomes.

## Introduction

Failures to replicate or reproduce published work have raised major concerns across several fields in science^1–3^. These reports have catalyzed both meta-research to explore the factors leading to diminished replication and reproduction rates^4–8^, and the launch of initiatives focused on improving these critical aspects of scientific research^9–11^. In particular, the field of functional neuroimaging has progressively intensified its efforts to address the challenges of replicability and reproducibility^12,13^.

A significant part of the problem in neuroimaging relates to the complexity of the analysis. As neuroimaging data analysis has grown increasingly complex over recent decades^14,15^, it has become essential to ensure transparency in reporting analysis details to support reproducibility^12,16–19^. Current analysis pipelines encompass multiple stages, including data selection criteria, preprocessing options, selection of data elements (e.g., regions of interest), subsequent post-processing options, and an array of statistical models for hypothesis testing, with a multitude of parameters for each of these stages^14^. This diversity in processing and analysis options provides considerable flexibility in analyzing the same dataset in various ways^15,16,19^, thereby enabling the exploration of a broader spectrum of research questions and accommodating different assumptions.

While analytical flexibility represents a significant advancement in the evolution of quantitative science, especially in handling high-dimensional data, it also poses challenges. Varying analysis pipelines can produce markedly divergent results, potentially leading to altered interpretations or even contradictory outcomes^15,20,21^. Analytical flexibility also leads to analytical variability: when researchers can select from a broad spectrum of possible and justifiable analytical options, they are likely to make different choices. This freedom diminishes the comparability of results between studies, and even within the same study^22–27^. Moreover, due to the extensive array of preprocessing and analysis options, researchers might be inclined to initially test pipelines aligning most with their expectations and exclusively report these outcomes. This approach, influenced by expectation and selection bias, results in undisclosed manipulation of analytical flexibility, ultimately undermining its reliability and credibility^13^.

Experiments conducted in collaboration with research communities have been instrumental in assessing the impact of analytical variability. In these studies, researchers are given the same dataset and test pre-specified hypotheses using analytical methods that they consider most suitable. Such experiments have been conducted with some neuroimaging techniques, including functional magnetic resonance imaging (fMRI) and electroencephalography (EEG), and they have shown that researchers use a wide array of analysis pipelines, which can lead to substantial differences in the reported findings and conclusions drawn from the same data^25,26,28–32^.

Functional Near-Infrared Spectroscopy (fNIRS) is another neuroimaging tool that has seen significant growth over the past decades. This optical imaging method utilizes near-infrared light to measure changes in brain hemoglobin concentrations^33–35^. The portability, ease of use, accessibility, and relatively low cost of fNIRS, compared to fMRI, highlight its substantial potential for routine use in scientific, clinical, and commercial settings. It is particularly advantageous in populations or experimental paradigms where fMRI is less suitable, for instance, in low-to-middle income countries, or for neurodevelopmental research on infants and children. This adaptability allows for more complex paradigms with high ecological validity and low restrictions on natural movements and behaviors, but can also introduce additional variability into data analysis. The quality of fNIRS signals can be influenced by hair and skin characteristics^36^, requiring tailored preprocessing and analysis approaches, which further adds to the variability across analysis pipelines. Moreover, the ongoing development of new hardware and analytical pipelines contributes to this variability.

Despite sharing similarities with EEG and fMRI, fNIRS has unique features and has been used in experimental designs that necessitate careful consideration regarding analytical flexibility. Directly applying conclusions from other techniques to fNIRS is not straightforward. As the adoption of fNIRS expands across various fields, research at the community level is needed to assess a) how analytical variability manifests across the spectrum of analytical choices and b) how these different choices affect analysis results and conclusions that can be drawn from identical fNIRS data. Initial investigations suggest considerable analytical variability across fNIRS pipeline settings^37–39^. However, a detailed analysis focusing on this variability within fNIRS has yet to be conducted.

Building upon previous investigations, the fNIRS Reproducibility Study Hub (FRESH) project explored the wide-ranging analysis methods employed within the fNIRS research community. By extending previous efforts, we quantified the variability in results when different researchers assessed the same dataset both at the group and individual levels. Given the complexity of experimental protocols and data analysis pipelines and the lack of standardization in fNIRS research^40,41^, understanding these variations is essential for progress. More importantly, addressing such variability in a period of increasing fNIRS significance in neuroscience is a crucial step toward establishing standards in the field.

For this purpose, this study invited members of the global fNIRS community to participate in a multi-lab analysis experiment. Researchers were provided with two fNIRS datasets, a brief description of the experimental protocols, and a list of pre-defined group-level and individual-level hypotheses related to research questions that could be investigated with these datasets. Researchers were assigned the task of testing these hypotheses. Importantly, researchers were not provided with instructions on how to perform these tasks other than based on their own expertise and best judgment.

Details of the experimental protocol and fNIRS acquisition are provided in the Methods section. The first dataset (Dataset I) comprised fNIRS recordings obtained from an auditory paradigm, which included speech, auditory noise stimuli, and silence, each lasting ~5 s. The hypotheses tested using this dataset were focused on group-level analyses:

H1. Speech stimuli evoke responses in the left Heschl’s gyri (HG);

H2. Speech stimuli evoke larger responses in the left HG than the noise stimuli;

H3. Speech stimuli evoke larger responses in the left HG than the right HG;

H4. Speech stimuli evoke response in the left inferior frontal gyrus (LIFG);

H5. Speech stimuli evoke larger responses in the LIFG than noise;

H6. Speech stimuli evoke responses in the occipital cortex;

H7. The silence condition evokes a response in the occipital cortex.

Among these, H1^42,43^ and H2 are strongly supported by existing literature^44,45^, while H7 is strongly negated. Hypotheses H3-H6 are expected to be true but have less robust support from prior studies^46–49^.

The second dataset (Dataset II) was derived from a motor experiment in which participants performed repeated right- and left-hand finger tapping for 2 and 3 s. The hypotheses for this dataset focused on individual-level analysis:

H1. Left-hand finger tapping (FT) of 2 s duration evokes a response in the contralateral primary motor cortex;

H2. Left- and right-hand FT of 2 s duration, conditions combined, evokes a response in the contralateral primary motor cortex;

H3. Left- and right-hand FT of 2 and 3 s duration, conditions combined, evokes a response in the contralateral primary motor cortex;

H4. Left- and right-hand FT of 3 s duration, conditions combined, evokes a greater response in the contralateral primary motor cortex than left- and right-hand FT of 2-s duration, conditions combined.

The first three hypotheses are strongly supported by existing literature, while support for H4 is less conclusive^50–53^.

After completing data analysis, researchers were asked to submit their results using a specifically designed form that inquired about the statistical outcomes for all pre-specified hypotheses. In a follow-up questionnaire, researchers were requested to provide further details about their analysis pipeline, including specifics about preprocessing and analysis steps they had performed.

In this work, we synthesize and interpret the diversity of analysis approaches within the fNIRS community. Briefly, we observed substantial variability in analysis pipelines, with no two teams using the same approach. Despite this, we found high agreement on group-level results, especially for hypotheses strongly supported by prior research. Agreement is lower for individual-level analyses but improves with better data quality. The main sources of variability across teams are linked to pruning choices, hemodynamic response function models, and the analysis space used for statistical inference. These findings reveal the extent and nature of analytical variability and its potential implications for research outcomes based on fNIRS.

## Results

Of the 223 researchers who initially registered for the study, 102 ultimately submitted their results. Eight researchers presented their work individually, while the rest formed 30 groups, resulting in 38 unique analysis submissions. Details from researchers’ backgrounds can be found in the Methods section.

### Hypotheses testing outcomes exhibit great variability across analysis pipelines

**Fig. 1:**
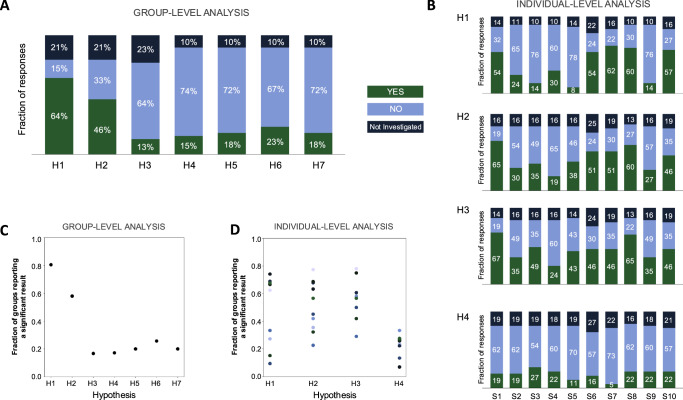
Variability in hypothesis testing results across teams. **A** Proportion of teams supporting each group-level hypothesis, H, in Dataset I (see text for the description of each hypothesis). **B** Percentage of teams supporting each individual-level hypothesis in Dataset II, separated by subject, S. In both panels, bar segments indicate the fraction of teams that supported (‘YES,’ green), rejected (‘NO,’ blue), or did not test (‘Not Investigated,’ navy), and the numbers in each bar represent the percentage of each response. **C** Proportion of teams reporting a significant result among those that tested the hypothesis in Dataset I (*n* = 31 (H1, H2), *n* = 30 (H3), *n* = 35 (H4-H7)). **D** Proportion of teams reporting a significant result among those that tested the hypothesis in Dataset II (*n* = 30–34 (H1, depending on the subject), *n* = 28–33 (H2, depending on subject), *n* = 28–38 (H3), *n* = 27–32 (H4)). Each color represents a different participant. (FT finger tapping, PMC primary motor cortex, LIFG left inferior frontal gyrus, HG Herschl’s gyrus).

Figure 1 presents the descriptive statistics depicting variability in hypothesis testing results among different groups. In the group-level analysis of Dataset I (Fig. 1C), at least 80% of the groups agreed on five out of seven hypotheses. Among these, three hypotheses (H1, H2, and H7) were strongly supported by existing literature. The outcomes from teams aligned with this literature support, with the highest agreement observed for the first two hypotheses (81% and 58%, respectively) and a strong consensus in rejecting H7 (80%). In contrast, hypotheses with weaker or inconsistent literature support exhibited lower agreement, with teams predominantly rejecting them despite their expected validity.

Upon conducting individual-level analysis using Dataset II, over 60% of the groups demonstrated agreement in all ten participant datasets for H1 and H4. For H2 and H3, a consensus was observed in seven and four out of ten participants, respectively (Fig. 1C). Although the percentage agreements for individual-level hypotheses were lower compared to the group-level hypotheses, they aligned with prior knowledge. The mean ± standard deviation of percentage agreement to the expected hypotheses outcomes for the first three hypotheses were 0.44 ± 0.26, 0.51 ± 0.19, and 0.54 ± 0.17, respectively, while H4 showed a lower agreement of 0.22 ± 0.07.

To assess potential systematic differences between participants in testing individual-level hypotheses, we calculated the mean signal-to-noise ratio (SNR) for each participant and categorized them into two groups: high SNR (participants 1, 2, 6, 7, 8, and 9) and low SNR (participants 3, 4, 5, and 10). We then computed the mean percent agreement for each hypothesis for both groups. For the high SNR group, the mean percent agreements were 52% for H1, 58% for H2, 59% for H3, and 21% for H4. For the low SNR group, the corresponding agreements were considerably lower: 31% for H1, 41% for H2, 47% for H3, and 24% for H4.

### Variability across different analysis pipelines is driven by both signal processing and statistical analysis choices

**Fig. 2:**
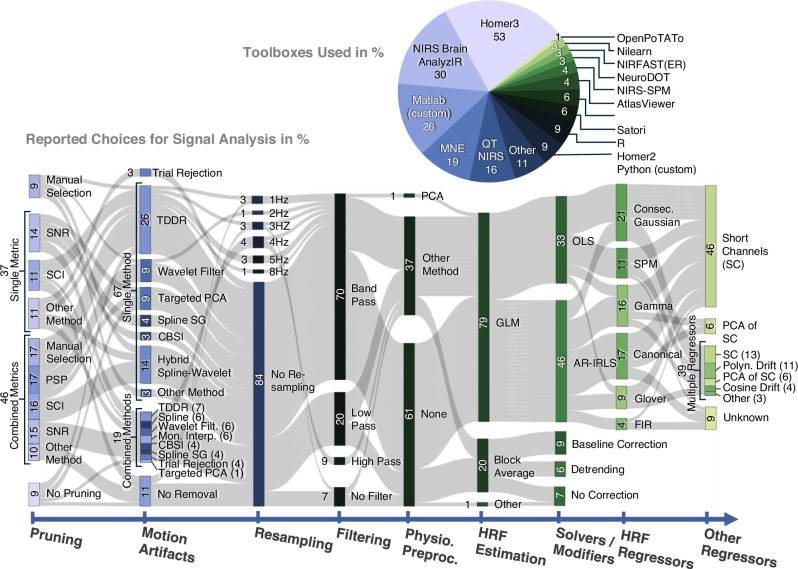
Overview of signal processing pipeline choices across teams. The teams’ pipeline choices for signal processing to extract the hemodynamic brain responses for subsequent statistical analysis are shown in a Sankey flow diagram with stages grouping choices by typical processing categories. All numbers are given in % rounded to two digits without decimal digits to improve readability. Multiple combined toolboxes in the analysis count individually toward each category (sum > 100%) in the pie chart. Processing stages: Pruning – method for identifying channels to drop from the analysis; (SCI) Scalp Coupling Index, (PSP) Peak Spectral Power, (SNR) Signal to Noise Ratio. Motion Artifacts – method for mitigating motion artifacts; (CBSI) Correlation Based Signal Improvement, (TDDR) Temporal Derivative Distribution Repair, (Spline SG) Spline interpolation with Savitzky-Golay filtering, (Mon. Interp.) Monotonous Interpolation. Resampling – resampling to a new sample rate for analysis. Filtering – temporal filtering. Physio. Preproc. – Other preprocessing *methods* for removal of physiological nuisance signals before HRF extraction. HRF Estimation – method for extraction/estimation of the hemodynamic brain response, GLM General Linear Model. Solvers/Modifiers – details for HRF estimation. (OLS) Ordinary Least Squares solution, (AR-IRLS) Autoregressive Iteratively Reweighted Least Squares. HRF Regressors – (only GLM) choice of regressors to model the hemodynamic response; (Consec. Gaussian) Consecutive Gaussians, (SPM) Statistical Parametric Mapping, (FIR) Finite Impulse Response. Other Regressors – (only GLM) choice of additional regressors to model physiology; (SC) Short Channels, (PCA of SC) First Principal Components of all Short Channels.

**Fig. 3:**
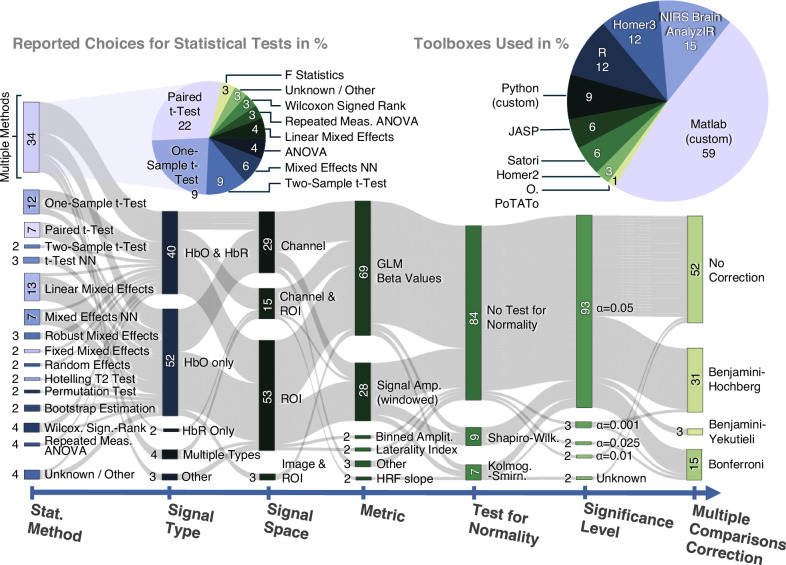
Reported statistical analysis steps for fNIRS data analysis pipelines. The teams’ pipeline choices to test the working hypotheses of this study are shown in a Sankey flow diagram with stages grouping choices by typical analysis categories based on *n* = 38 pipelines for each of the two datasets. Multiple combined toolboxes in analysis count individually to each category (sum > 100%) in the pie chart. Statistical stages: Stat. Method – Statistical method employed for hypothesis testing. (t-Test NN) t-Test without further specification of type. (Mixed Effects NN) Mixed Effects model without further specification of type. Signal Type – tests performed on brain responses measured via HbO, HbR, both, or other. All numbers are given in % rounded to two digits without decimal digits to improve readability. Signal Space – tests performed on responses from individual channels, in image space, or for regions of interest (ROI). Metric – tests performed on GLM beta weights, windowed signal amplitude, or other options. Test for Normality – no or if yes, which one; (Kolmog.-Smirn.) Kolmogorov–Smirnov Test. Significance Level – Threshold for statistical significance. Multiple Comparison Correction – none or three different approaches.

To better comprehend the influence of analytical choices on the variability of the outcomes for hypothesis testing, we examined the choices made at various stages of fNIRS data analysis. Typically, these stages contain preprocessing methodologies such as motion artifact correction, pruning and filtering before extraction of the hemodynamic responses and statistical testing. The detailed choices in preprocessing (Fig. 2) and statistical testing (Fig. 3) are visualized using Sankey diagrams.

The Sankey diagrams illustrate the global distribution and heterogeneity of choices along the multi-staged analysis pipelines. We grouped pipeline stages that belong to a typical category of fNIRS methods, regardless of the order they appeared in the analysis. The resulting diagrams show the absolute frequency of each method employed across all submitted analysis reports. In some stages, researchers reported combining multiple procedures (e.g., Scalp Coupling Index (SCI) and Peak Spectral Power (PSP) for joint channel quality assessment). In the diagrams, these multiple choices are grouped (merged vertically) and labeled accordingly with a square brace. To improve interpretability within these groups, reported numbers are given as absolute frequencies for each method across all submitted reports, not their relative frequency within the group. For example, 46% of all reports used multiple combined methods for channel pruning, and 37% used only one method; 16% of all reports used the SCI combined with other metrics, and an additional 11% of all submissions used the SCI as the only metric. Therefore, SCI was applied as a method in 27% of all reported pipelines, and 17% of all reports used the PSP as a metric for pruning, however, only in combination with other metrics and never alone.

Homer3, AnalyzIR, and MNE were the most frequently used general-purpose fNIRS toolboxes for analysis, and Quality Testing of Near Infrared Scans (QT-NIRS) was the most-commonly used toolbox for quality assessment, specifically for channel pruning. Approximately a quarter of all researchers utilized in-house custom MATLAB scripts for their analyses.

The majority of groups reported pruning poor-quality channels (91.4%) and mitigating motion artifacts (88.6%). For this, a wide range of approaches was used, leading to high variance in these pipeline stages. Regarding the hemodynamic response due to stimulation, about 79% of analyses were performed using a general linear model (GLM), for which a slight majority used the Autoregressive Iteratively Reweighted Least Squares (AR-IRLS) method for solving the model rather than an ordinary least squares (OLS) solution. About 71% of those who used a GLM also employed short channels or the principal component of short channels as physiology regressors - either alone or in combination with other regressors. It is also noteworthy that 20% of all analyses were performed using block-averaging, i.e., without any physiological model for the HRF extraction.

Figure 3 visualizes the reported choices for the hypothesis testing and statistical analysis. MATLAB, R, and Python were the most frequently used languages for statistical analysis, and Homer3 and NIRS Brain AnalyzIR were the top cited toolboxes. It is worth noting that the largest heterogeneity in the statistical analysis pipeline is in the reported choices of methods employed for statistical testing. About 34% of all groups reported using/combining multiple different statistical methods. Approximately 72% of the groups reported using a test of the *t*-Test family, and 37% used a type of mixed effects model. Most researchers (52%) chose to infer brain activation using only HbO, while 40% used both HbO and HbR signals. Statistical analyses were performed in channel or ROI spaces for most cases (only 3% reported analysis of ROIs in image space). 69% of all statistical tests were performed on the estimated HRF beta weights from GLM analysis, and less than one third on windowed signal amplitudes or other metrics. Most groups (84%) did not test for normality and used a significance threshold of α = 0.05 (93%). Only 48% of all groups reported corrections for multiple comparisons in their analyses.

**Fig. 4:**
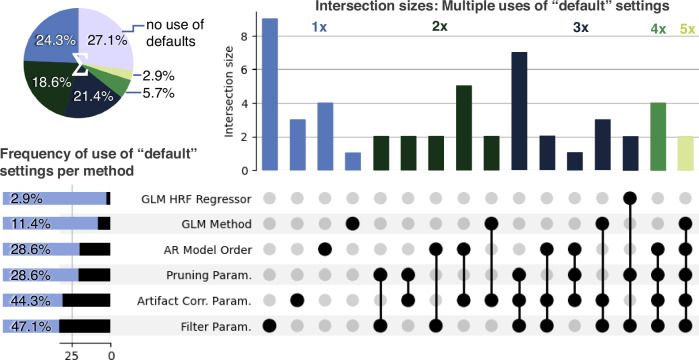
Reported use of toolbox default settings across analysis pipelines. UpSet plot showing the use of default parameters and settings in the groups’ analysis pipelines based on n = 38 pipelines for each of the two datasets. Rows display individual categories for which default settings could be chosen, and horizontal bar plots their cumulative frequency (e.g., groups chose default filter parameters in 47% of all reported analyses). The pie chart shows the fraction of groups that used default settings in 1, 2, 3, 4 and 5x categories (matching the color code of the intersection size bars, e.g. 2.9% used defaults for 5 categories). Connected black dots in columns display intersection (combination) of categories and vertical bar plots the frequency (intersection size) of these combinations. For example, three groups reported using default settings for the GLM method, artifact correction, and filter parameters combined, and four groups reported using the default settings only for the AR Model Order.

Aside from the selection of methods for analysis, the choice of parameters for each method plays an important role, as they can significantly alter analysis outcomes. Figure 4 summarizes how often groups reported choosing “default settings/parameters” provided by the analysis toolboxes and at which analysis step. About 27% of all analyses were performed by groups manually configuring all parameters, i.e., without using default settings in their pipeline. About 50% of groups reported using default parameters for their filter settings and artifact correction methods, and a bit under a third of the groups used default parameters for signal quality/pruning methods. Almost a third of all groups used default settings in three or more signal-processing steps. A post-hoc analysis did not show any significant correlation between the frequency of using default values and the researcher’s self-reported confidence level in their results or analysis skills.

### Choice of Analysis Pipeline Affects Hypothesis Testing Outcomes

We investigated the effect of the choice of the major analysis steps on the resulting hypothesis testing outcomes across teams using a logistic regression approach. The results reveal differential impacts of these processing steps on the logistic regression fit, with significance determined at a threshold of *p* < 0.05. Table 1 summarizes the number of hypotheses for which each distinct category under each analysis step significantly explains the variation in hypothesis testing outcomes across teams. Notably, for the HRF Estimation step, “Block Averaging” emerged with nine significant contributions, considering “GLM” as the reference category. Similarly, for the HRF regressor, “Flexible” and “None” (i.e., block averaging, which does not have any HRF regressor), each emerged with four significant contributions, with “Fixed” as the reference category. (Please note that, for the regression analysis, we merged the HRF regressor categories “Consecutive Gaussians” and “FIR model” into a single category called “Flexible”, and “Gamma,” “Canonical,” “SPM” and “Glover” into a single category called “Fixed”.) For the Signal Space, the choice of “Channel” exhibited nine significant contributions, with “ROI” as the reference category. The “Combo” category under “Pruning” demonstrated three significant contributions, while the “Visual” category yielded four. “Multiple Comparisons Correction” yielded two contributions for both “Benjamini–Hochberg” and “Bonferroni”, with “No Correction” as the reference category. For the “Motion Artifact Correction”, “Filtering,” and “Statistical Method” steps, no category showed a significant impact. After applying the Benjamini–Hochberg correction for multiple comparisons^54^ to adjust for the false discovery rate, none of the p-values from the 47 independent logistic regression analyses were found to be statistically significant.

**Table 1:** Pipeline factors significantly influencing hypothesis testing across teams

Pruning	# of H	HRF regressor	# of H	HRF estimation	# of H	Signal space	# of H	Multiple comparisons	# of H
Auto	REF	Fixed	REF	GLM	REF	ROI	REF	None	REF
Combo	3	Flexible	4	Block Averaging	9	Channel/ROI	0	Benjamini–Hochberg	2
Visual	4	None	4			Image/ROI	0	Bonferroni	2
None	1					Channel	9	Benjamini–Yekutieli	0
						None	0		

The number of hypotheses (among the total 47 hypotheses [7 group-level and 40 individual level]) that any given analysis pipeline step significantly contributed to the variance of results across teams (*p* < 0.05). The analysis steps were modeled separately.

To see the overall effect of each step on hypotheses outcomes, we first tested for any multicollinearity between the processing pipeline steps that explain the variability in the hypothesis testing outcomes. We found a significant association between the HRF Estimation Method and Pruning (chi-squared statistic = 8 and *p* = 0.04), HRF regressor (chi-squared statistic = 30 and a *p* < 0.001) and Multiple Comparisons Correction (chi-squared statistic = 16 and a *p* = 0.001). Excluding these three due to the found associations, we then performed a multiple logistic regression, including the HRF estimation method and Signal Space as independent variables in the model. “Block Averaging,” when contrasted with “GLM” as the reference category, yielded five significant outputs, and “Channel” against “ROI” as the reference category yielded five significant outputs when all categories under HRF Estimation Method and Signal Space were jointly included in the regression model.

**Fig. 5:**
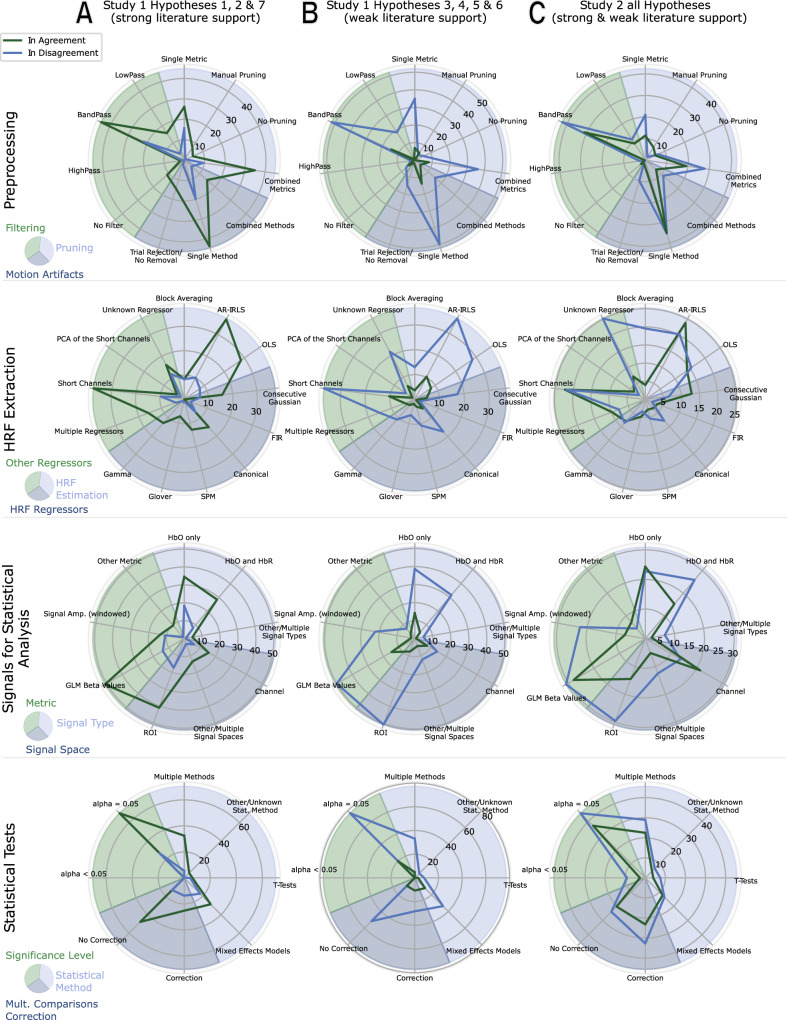
Distribution of pipeline choices by agreement with literature support. Radar Charts of the distribution of choices in the analysis pipelines based on their (dis-)agreement with expected hypotheses outcomes, grouped by different pipeline stages based on *n* = 38 pipelines. **A** Results for Hypotheses 1, 2, and 7 of Dataset I, which have been supported by prior literature and therefore have a high expectation of being confirmed. **B** Results for Hypothesis 3–6 of Dataset I, which are expected to be true but are only weakly supported by prior literature. **C** Results for all four hypotheses analyzed at the individual level in Dataset II. In all cases, radial axes numbers represent joint probabilities of pooled individual hypothesis outcomes for a chosen category among all users in percent.

Figure 5 compares choices in pipelines based on agreement or disagreement in hypothesis outcomes, grouped by the strength of prior literature support (i.e. strong or weak literature support). The differences in the distributions of methodological choices based on agreement or disagreement with expected hypothesis outcomes visually complement the findings from our logistic regression analysis. For instance, variations are observed in the selection of HRF estimation methods and regressors (Fig. 5, Row 2), the choice of signal space (e.g., using ROI; Fig. 5, Row 3), and the application of multiple comparisons correction (Fig. 5, Row 4).

In particular, where these differences are most pronounced, an inverse trend emerges between agreement and disagreement for hypotheses with strong literature support versus hypotheses with weak literature support. For instance, in the case of HRF extraction (Fig. 5, Row 2, Column 1), 46% of researchers used the AR-IRLS method (refer to Figs. 2 and 3 for total numbers). Among these, the testing outcomes for hypotheses with strong literature support (H1, H2, and H7) aligned with expected results more than three times as often (36%) as outcomes that disagreed with expectations (10%). Conversely, for hypotheses with weak literature support, the majority of AR-IRLS users reported outcomes in disagreement with expectations. This trend aligns with AR-IRLS being recognized as a statistically robust approach, offering superior control over false positives^40,55^.

### The consistency of hypothesis testing outcomes is higher among researchers with high self-reported confidence

**Fig. 6:**
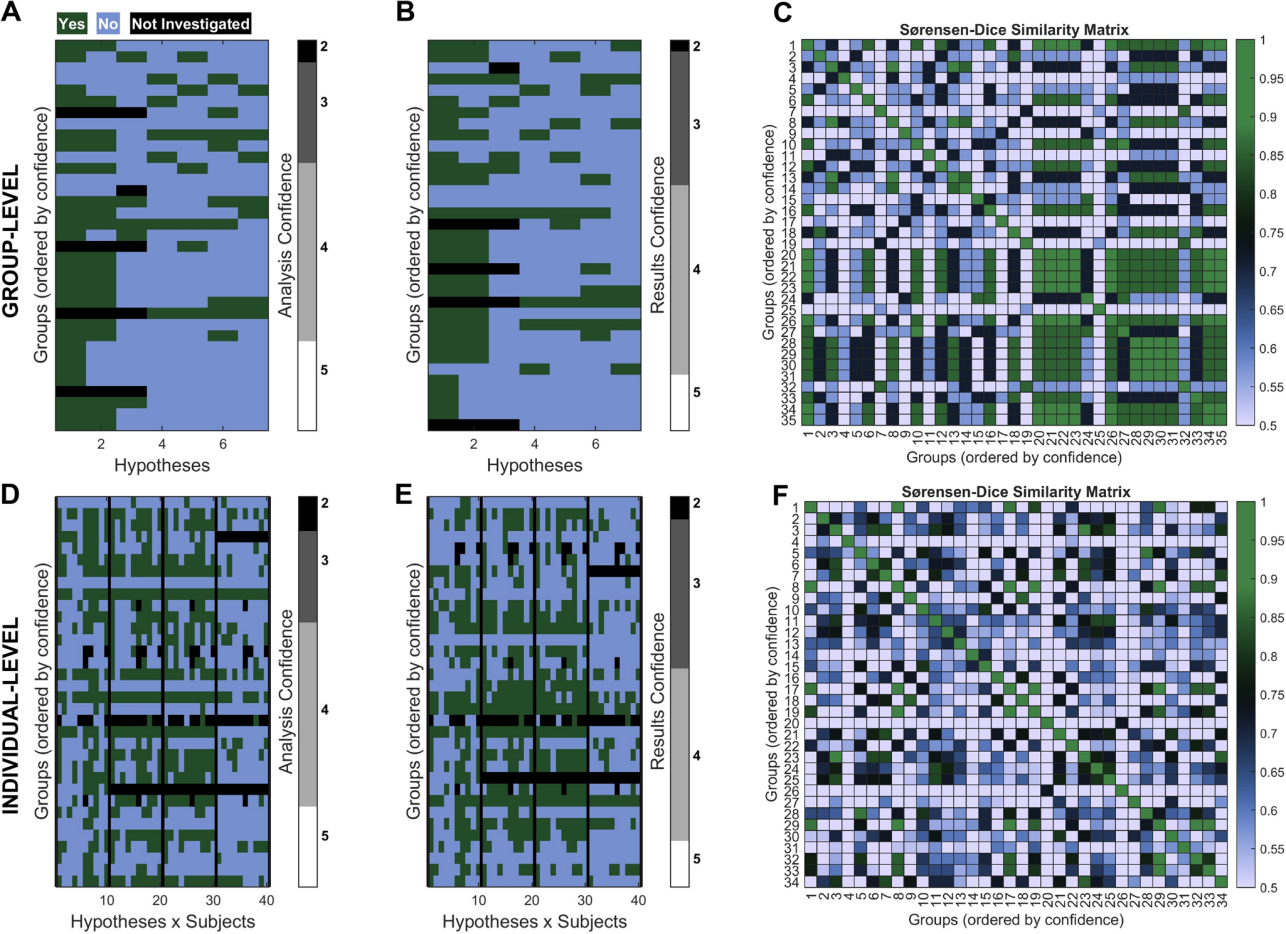
Relationship between hypothesis testing outcomes and self-reported confidence. Panels **A**–**C** represent group-level hypotheses, while panels **D**–**F** illustrate individual-level hypotheses. Hypothesis testing results grouped by teams’ self-reported confidence in their analysis skills presented for group-level hypotheses (**A**) and individual-level hypotheses (**D**). Same results grouped by confidence in the reported outcomes presented for group-level hypotheses (**B**) and individual-level hypotheses (**E**). Sørensen-Dice similarity matrices illustrating the consistency of hypothesis outcomes across teams, organized according to self-reported confidence in their analysis skills presented for group-level hypotheses (**C**) and individual-level hypotheses (**F**). The colorbar represents the Sørensen-Dice coefficient values, ranging from 0.5 to 1. Please note that not all groups reported confidence; hence the number of groups in this plot is smaller than the total number of groups, which is 38.

To explore potential factors influencing variability in hypothesis outcomes, we also tested several specific hypotheses beyond our primary research questions. Figure 6 displays the hypothesis testing results with respect to the groups’ self-reported confidence both in their analysis skills and in their reported results for the group-level (Fig. 6A, B) and individual-level hypotheses (Fig. 6D, E). In addition to the hypothesis testing results, Panel C illustrates Sørensen-Dice Similarity matrices arranged according to the groups’ self-reported confidence in their analysis skills, providing a visual representation of how different levels of self-reported confidence correspond to the similarities between the hypothesis testing outcomes (Fig. 6C, F). Notably, the Sørensen-Dice Similarity matrix for the group-level analysis shows increased similarity with increasing self-reported confidence in analysis (Fig. 6C). Table 2, summarizing Fig. 6C, presents self-reported confidence levels and the corresponding Sørensen-Dice coefficients for Dataset I (group-level analysis) and Dataset II (individual-level analysis). The average Sørensen-Dice coefficient for group-level Analysis (Mean ± SD: 0.59 ± 0.24, *n* = 595) was significantly higher than the one for individual-level Analysis (Mean ± SD: 0.52 ± 0.20, *n* = 561) (two-sided unpaired *t*-test, *t* = 5.7, *p* < 0.001, Cohen’s d = 0.34, 95% CI [0.049, 0.100], *p* < 0.001). In Dataset I (group-level analysis), higher self-reported confidence levels (4 and 5) demonstrated improved Sørensen-Dice coefficients. For Dataset II (individual-level analysis), the Sørensen-Dice coefficients varied slightly across self-reported confidence levels, with self-reported confidence level 5 showing the highest coefficient (Mean ± SD: 0.58 ± 0.16). Two-sided unpaired *t*-tests performed to compare similarity values obtained from Sørensen-Dice analysis between the sets of self-reported confidence levels {2, 3} and {4, 5} yielded a significant difference for the group-level analysis (Mean ± SD (Low Confidence): 0.50 ± 0.20, *n* = 37; Mean ± SD (High Confidence): 0.61 ± 0.27, *n* = 148; *t* = −2.5, *p* = 0.015, Cohen’s d = −0.45, 95% CI [−0.209, −0.023]) but not for the individual-level analysis (Mean ± SD (Low Confidence): 0.52 ± 0.19, *n* = 31; Mean ± SD (High Confidence): 0.48 ± 0.20, *n* = 141; *t* = 1.0, *p* = 0.32, Cohen’s d = 0.20, 95% CI [−0.039, 0.118]).

**Table 2:** Agreement in results as a function of analysis confidence

	Dataset I	Dataset II
Confidence	Group-level analysis (mean ± *SD*)	Individual-level analysis (mean ± *SD*)
2	0.57 ± 0.00	0.50 ± 0.21
3	0.49 ± 0.20	0.52 ± 0.19
4	0.57 ± 0.27	0.46 ± 0.20
5	0.80 ± 0.16	0.58 ± 0.16
Average	0.59 ± 0.24	0.51 ± 0.20

Mean and standard deviation (*SD*) of the Sørensen-Dice Similarity for different self-reported confidence in analysis skills.

Further analysis revealed differences in the percent agreement with the expected outcomes between teams with low self-reported confidence (levels {2, 3}) and high self-reported confidence ({4, 5}) across the group-level hypotheses, particularly for those with strong support from the literature. For H1, which has strong literature support, teams with high self-reported confidence showed 90% agreement, compared to 60% for teams with low self-reported confidence. For H2, which also has strong literature support, high-confidence teams achieved 67% agreement, while low-confidence teams reached 40%. For H7 (expected to be false according to literature), 83% of high-confidence teams disagreed with the hypothesis, compared to 73% of low-confidence teams.

Both self-reported confidence in analysis skills and in results were significantly correlated with the average number of years of fNIRS experience among team members. Specifically, confidence in analysis skills correlated with experience (*r* = 0.36, *p* < 0.002, 95% CI [0.14, 0.55], *n* = 69), as did confidence in results (*r* = 0.27, *p* < 0.027, 95% CI [0.03, 0.47], *n* = 69). These n values reflect combined group-level and individual-level data, including only groups that reported confidence ratings.

Furthermore, there was a statistically significant positive correlation between confidence in analysis skills and confidence in the resulting outcomes (*r* = 0.62, *p* < 0.001, 95% CI [0.44, 0.74], *n* = 69 (group-level and individual-level combined, including only those groups that reported confidence)), indicating that teams who felt more confident in their analytical abilities also tended to exhibit greater confidence in their results.

### Greater hypothesis outcome agreement is associated with specific analytical practices

We have also examined the methodological choices across different research groups, focusing on those with high similarity in hypothesis outcomes compared to the rest. Our findings revealed notable differences in approaches to data analysis, particularly in pruning, artifact rejection, and the use of General Linear Models (GLM), which may contribute to the variability in fNIRS data interpretation.

Pruning strategies varied significantly; no groups with high Sørensen similarity scores used Signal-to-Noise Ratio (SNR) as their sole quality/pruning metric, preferring to combine it with other metrics. Conversely, 21.7% of groups with low Sørensen scores relied solely on SNR. Groups with high Sørensen scores were more likely to employ multiple channel quality metrics compared to their low-score counterparts (61.5% vs 34.8%). Conversely, manual selection for pruning was exclusively observed in the low Sørensen group. In terms of artifact rejection, only one group from the high Sørensen subset applied whole trial rejection, indicating this practice is not widespread.

The application of General Linear Models was more common among the high Sørensen groups than the low (92.3% vs 78.3%), with a corresponding decrease in block averaging usage. Regarding hemodynamic response function (HRF) regressors, a significantly larger portion of high Sørensen groups utilized a Gamma HRF regressor compared to their low Sørensen counterparts (30.8% vs 4.3%).

Our analysis revealed that a larger percentage of high Sørensen groups employed various short-separation regression methods (77.0% total; distribution: 46.2% short-separation only, 15.4% short-separation combined with other regressors, 7.7% PCA of short-separation combined with other regressors, and 7.7% PCA of short-separation only) compared to low Sørensen groups (69.5% total; distribution: 47.8% short-separation only, 13.0% short-separation combined with other regressors, 4.3% PCA of short-separation combined with other regressors, and 4.3% PCA of short-separation only). This demonstrates the high Sørensen groups’ inclination towards incorporating short-separation measurements in their analysis to potentially reduce confounding factors.

## Discussion

Noninvasive neuroimaging techniques have revolutionized our understanding of the human brain, opening up new avenues in various scientific fields. As technology has advanced, facilitating more flexible experimental protocols, it has also increased variability in data analysis, affecting the consistency of conclusions drawn from neuroimaging techniques, as seen with fMRI and EEG.

Our results show that fNIRS faces similar challenges. Over the past decade, fNIRS has become a reliable and widely adopted tool^35^, particularly in experiments where other techniques fall short, due to its flexibility and lower operational threshold compared to fMRI. However, while it is easy to acquire interpretable signals, assessing their meaningfulness remains difficult. In addition, rapid technological advances have outpaced the standardization of data analysis, making it crucial to understand how different analysis pipelines impact scientific consistency. The results presented in this work shed light on this issue and provide insights for other neuroimaging techniques by exploring the influence of analysis flexibility and other key factors that contribute to variability in outcomes, such as researcher confidence.

Overall, we observed substantial variability across teams in hypothesis testing, particularly at the individual level. It is important to note that, because the data originated from different sources, any direct comparison between group- and individual-level agreement is prone to bias from confounding factors such as variations in data quality. Despite this limitation, higher agreement rates (>80%) were seen in most group-level hypotheses (5 out of 7 hypotheses), indicating more consistent outcomes when individual variations are treated as noise or measurement error.

Notably, the high percentage of agreement among teams for hypotheses with strong literature support reinforces the potential of fNIRS as a reliable neuroimaging technique, especially in the context of group-level analysis grounded in well-supported research contexts. As the strength of evidence for a hypothesis correlates with the contrast-to-noise ratio (CNR), these results imply that higher expected CNRs tend to result in greater agreement among teams, regardless of differences in the analysis pipelines used for fNIRS group-level studies.

In contrast, individual-level hypotheses showed lower overall agreement, with only two hypotheses achieving levels of agreement comparable to those observed at the group level, and in only two out of 10 participants. This discrepancy likely arises from the greater sensitivity of individual-level analyses to intrinsic differences in anatomy and physiology, which makes individual outcomes more dependent on specific choices in the analysis pipeline (e.g., exclusion criteria resulting in the removal of different trials and/or fNIRS channels across subjects).

Despite the lower agreement observed in individual-level analyses compared to group-level analyses, the degree of agreement among groups consistently mirrored the strength of literature support for each hypothesis, with weak hypotheses resulting in lower agreement. Importantly, individual-level analyses revealed a clear effect of data quality (assessed by SNR) on the consistency of hypothesis testing outcomes: participants with higher SNR yielded greater agreement in their results. While this aligns with the expectation that higher-quality data produce more reliable outcomes, it is important to acknowledge that many factors influence SNR in fNIRS, including those related to population diversity and specific physiological or anatomical characteristics. These challenges are particularly relevant when collecting data from globally representative populations, where achieving high SNR can be more difficult. In our study, the limited number of participants in Dataset II constrained our ability to statistically examine the relationship between participant demographics, SNR, and outcome agreements. Nonetheless, the observed impact of data quality on reproducibility does not diminish the importance of advancing equity and inclusion in neuroimaging research, and further highlights the need to develop more robust data analysis methods and increase sample size.

No two teams adopted identical workflows, echoing findings from a previous study on variability in fMRI^25^. The flexibility in choosing analysis pipelines led to considerable differences in the methods used, particularly in signal preprocessing and statistical testing. Recent efforts to establish best practices aim to enhance reproducibility, particularly for these stages^40^. For instance, the impact of confounding systemic signals on fNIRS is well-recognized, along with the importance of methodologically addressing these by including additional systemic information in the analysis whenever possible, such as short-channel measurements as proxies for systemic physiology, which can greatly reduce false discovery rates^56,57^. Many groups (79%) used GLM for regression of systemic signals, while only a few (20%) used less robust methods like block averaging, which does not account for systemic physiological effects.

Among GLM users, a slight majority (46% versus 33%) opted for AR-IRLS over OLS, as it effectively reduces false positives by accounting for the intrinsic temporal correlation of fNIRS signals through autoregressive models with optimal order selection^58^. Choices for the HRF regressor varied across six variants, from those allowing extensive shape fitting like the FIR model, to those with minimal parameters such as the Canonical model. This variety highlights the need to establish a consensus on selecting HRF regressors. Regarding the additional regressors to the HRF, 70% of the groups that employed GLM used short-channel-based information.

It is also worth noting that the majority of groups applied temporal smoothing (90%) to the fNIRS signal, using band-pass (70%) or low-pass (20%) filtering. Therefore, these filter weights need to be accommodated in the estimation of temporal correlation with GLM^40,58^. Ignoring these correlations can lead to biased estimates of degrees of freedom and standard error, thereby invalidating statistical tests.

A slight majority (52%) used HbO for statistical testing, as it generally shows a stronger response than HbR, though it is more susceptible to task-related systemic physiological changes^39^, potentially leading to a higher false discovery rate. Groups also varied in their approach to defining the signal space for statistical testing, with 53% using Regions of Interest (ROIs) to reduce spontaneous noise and improve reliability, while 29% conducted their analyses at the channel level, which can increase sensitivity to spatial variability, especially in studies with low-density fNIRS setups lacking detailed spatial information. Pre-experiment optode registration can reduce this issue by ensuring consistent placement of optodes, thus enhancing reproducibility^59^. When anatomical registration is properly performed, translating data from the channel space to either the ROI or voxel spaces generally results in less variability^60,61^. A small amount (15%) utilized a combination of both ROI and channel-based methods.

Additionally, 52% of all groups did not perform multiple comparisons correction, increasing the risk of false positives^56,57^. The statistical tests of this study were based on measurements from multichannel fNIRS systems (e.g., 64 source-detector combinations, or channels, were used for the motor dataset. When a significance threshold of uncorrected *p* = 0.05 was applied, on average, 5% of the 64 channels (i.e., 3 channels) could be false positives). The number of false positives increases even further when using voxels in the case of reconstructed image space from diffuse optical tomography or interpolating kernels. This issue, referred to as the multiple testing problem, can be addressed using methods that control the family-wise error rate at level α (e.g., corrected *p* = 0.05), such as Benjamini–Hochberg, Bonferroni correction, or random field theory^62^. These methods calculate the probability that t-values exceed a given threshold across a set of channels, voxels, or clusters.

Our logistic regression analysis revealed that variability in hypothesis testing outcomes was partially explained by the choices at different stages of data processing, particularly methods for data pruning and the choices for HRF estimation, HRF regressor, signal space for statistical testing, and the use of multiple comparisons correction. Specifically, opting for block averaging rather than a GLM in HRF estimation contributed to the variability in the hypothesis outcomes. Similarly, the choice of performing statistical testing on individual channels over ROIs explained some of the variability in the hypothesis outcomes.

The broader utilization of GLM as an HRF estimation method among the groups that showed high similarity in hypothesis testing outcomes and who, at the same time, has longer experience in the field, indicates a preference for robust statistical frameworks that may better accommodate the confounding signals in the fNIRS data. The inclusion of short-separation (SS) regression techniques by a significant majority of this group further supports this. The pronounced difference in the use of Gamma HRF regressors between this group and the rest of the groups (30.8% vs 4.3%) points towards a methodological divergence in addressing hemodynamic response modeling.

When we break down the hypothesis testing results by self-reported confidence, higher confidence was associated with more consistent results at the group level. Confidence was also significantly correlated with the average number of years of fNIRS experience of the team members, suggesting that lower variability in hypothesis testing outcomes may be due to fewer errors in analysis pipelines and/or convergence towards certain analysis methods as experience in the field grows.

Interestingly, teams with greater confidence demonstrated better alignment with expected outcomes, particularly for well-supported hypotheses, highlighting the role of experience and self-assurance in achieving more reproducible outcomes. However, while confidence is often linked to higher decision-making accuracy^63^, we cannot conclusively attribute these observed consistencies to result accuracy without further validation, such as using a synthetic dataset with known ground truth.

It is worth noting that a few aspects of our study design should be considered before generalizing the findings above. First, the recruitment of researchers was based on an open call through conferences and social media, and while the sample was diverse and global, certain regions, particularly Asia and Africa, were underrepresented. Second, the datasets used in this study were chosen based on their availability and adherence to current best practices in fNIRS acquisition. Both datasets were collected in healthy, young adults using commercial continuous-wave fNIRS devices from the same manufacturer. While this choice helped control for some sources of variability, it limits the generalizability of our findings to datasets collected in other populations (e.g., infants, elderly) or acquired with other devices or fNIRS modalities, such as time-domain (TD) or frequency-domain (FD) fNIRS. For instance, TD-fNIRS allows for selective sensitivity to deeper regions^64,65^, while FD-fNIRS incorporates phase information that can enhance sensitivity and specificity in functional neuroimaging^66,67^. These modality-specific features could influence certain steps in the analysis pipelines discussed in this work.

Regarding the experimental paradigm, we selected the finger-tapping task for its standard use in fNIRS, ensuring familiarity among researchers and offering little novelty from a neuroscience perspective. Similarly, auditory stimulation is a primary brain function and is expected to yield more consistency than cognitive tasks, for example, even though it is less common in fNIRS. However, both datasets used a similar block design with short stimulation times and had been previously published, which may have introduced anchor bias, despite instructions not to consult the original results. That said, our hypothesis-driven framework, with predefined regions of interest, minimized the risk of double-dipping (i.e., the inappropriate reuse of data for both ROI selection and hypothesis testing)^68^. We found no evidence of such practices in the submitted reports. While this approach reduced the risk of circular analysis, it also limited our ability to assess how frequently such methodological pitfalls occur within the broader fNIRS community. The short task durations also resulted in lower SNR than most traditional fNIRS studies, potentially affecting pipelines differently and contributing to dataset-specific variability.

Overall, these design choices were intended to minimize other sources of variability unrelated to analysis pipelines, as more complex paradigms might have introduced additional variability or limited the number of researchers able to participate. This focus on simplicity, while suitable for our goals, limits the exploration of more advanced approaches, such as the ones employing multimodal analysis and artificial intelligence. These emerging methods represent exciting avenues for fNIRS research in the coming years and may even help standardize pipelines.

Lastly, the formulation of the hypotheses might have influenced analytical choices. Although we aimed to provide clear, objective hypotheses for testing and offered an open forum for clarifications, varying interpretations across groups could lead to differences in testing and reporting.

Taken together, our findings suggest substantial levels of analytical variability in fNIRS, moderated by researchers’ experience and confidence. Researchers use the degrees of freedom available in analyzing complex, high-dimensional fNIRS datasets, some of which were identified only after specifically querying them in a follow-up questionnaire. These results highlight the need for greater transparency and support for the adoption of open-science practices to enhance reproducibility and replicability in fNIRS research^19,40,41^. These efforts can reduce variability and enhance reproducibility, particularly in typical functional neuroimaging protocols^1,69^. However, it is important to recognize that consistency and reproducibility in outcomes do not equate to accuracy, as consistent results do not automatically reflect “ground truth”. Moving forward, future efforts should shift from describing variability to assessing the accuracy of data analysis pipelines and evaluating how different processing steps influence how closely the results align with expected outcomes.

While standard approaches and best practices have their place, it is important to also recognize the value of methodological diversity that converges to similar outcomes in the hypotheses studied. This emphasizes the robustness of fNIRS to some of the flexibility available in data analysis, which is beneficial for the field. In addition, encouraging a range of analytical pipelines allows for innovation and flexibility in addressing unique challenges posed by different research questions. Data and code sharing should further be promoted to allow researchers to reproduce published work or test new methodologies. While tools to do so are increasingly available^19^, more community efforts are required for their successful, widespread use, along with appropriate training opportunities at various levels to help researchers effectively and responsibly utilize these tools.

Moreover, global data pooling initiatives such as consortia would greatly increase possibilities for meta-analytical and mega-analytical research. Global consortia such as ENIGMA (Enhancing NeuroImaging Genetics through Meta Analysis) have become the gold standard in other fields such as clinical MRI research^70,71^. Consortia can aggregate large sample sizes and test and standardize data-quality control procedures. They yield robust, generalizable results, which has also motivated other imaging fields such as EEG to form consortia^72^. Similar developments would be desirable for fNIRS research^40,41^.

Adopting any or all of these tools requires a cultural shift in the fNIRS community that must be supported by key stakeholders, including academic societies and institutions, through educational initiatives within the community^1,73^. Without such collective effort, the full potential of these advancements cannot be achieved.

## Methods

### Participating researchers

A total of 223 researchers registered to analyze the fNIRS datasets. Ultimately, 102 researchers (55 female) submitted their results. Eight researchers submitted their work individually, while the remainder formed 30 groups with two to eight researchers each, resulting in 38 unique analysis submissions (35 unique submissions per study, as groups could submit results for either study or both).

The 38 submissions originated from 40 independent institutions worldwide, including nine different countries in Europe (accounting for 52.5% of the total submissions), the USA (20%), Canada and the Middle East (7.5% each), Asia and Latin America (5% each), and Australia (2.5%). Of the 30 groups that submitted their analysis, 27% (8 groups) participated in inter-institutional collaborations, while the rest of the groups were composed primarily of researchers from the same institution. In addition, 59% of the groups included at least one member who reported being an expert in fNIRS.

**Fig. 7:**
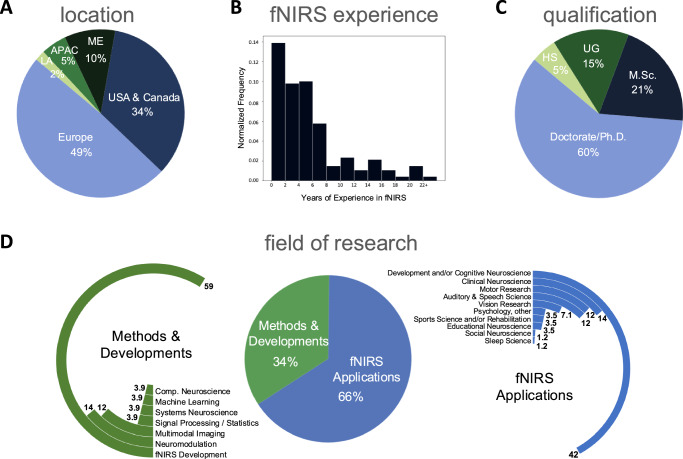
Demographic profile of participating researchers. A total of 102 researchers, grouped in 38 teams, submitted reports for analysis. Plots show their **A** geographic affiliation, **B** years of experience in fNIRS, **C** highest education qualification, and **D** self-reported fields of study. (LA: Latin America, APAC: Asia and Pacific, ME: Middle East, HS: High School, UG: Undergraduate).

Regarding the demographics of the 102 researchers (Fig. 7), nearly half (49%) were affiliated with institutions in Europe, followed by 34% in the USA or Canada. Researchers from institutions in the Middle East comprised 10%, while those from the Asia-Pacific region accounted for 5%, and 2% were associated with Latin American institutions.

Considering fNIRS experience, the researchers reported an average of 5.7 years (median = 4.0 years, standard deviation = 5.1 years), with a range spanning from novice (0 years) to highly experienced (25 years). The majority (60%) of these researchers had earned a Doctorate, 21% held a Master’s degree as their highest academic qualification, and 15% had completed an undergraduate degree. In addition, less than 5% of the researchers were undergraduate students.

Regarding their primary research focus, 66% of the researchers reported using fNIRS for applied research. These researchers reported working in a range of disciplines and application fields, including developmental and/or cognitive neuroscience (42%), clinical neuroscience (14%), motor processing (12%), auditory and speech processing (12%), visual processing (7.1%), sport science and rehabilitation (3.5%), educational neuroscience (3.5%), other topics in psychology not mentioned above (3.5%), sleep science (1.2%), and social neuroscience (1.2%).

The remaining 34% of researchers reported being primarily focused on developing neuroimaging data-analysis methods and/or instrumentation. Their areas of specialization included fNIRS methods and data analysis (59%), development of neuromodulation strategies involving fNIRS (14%), multimodal imaging (12%), signal processing and biostatistics (3.9%), systems neuroscience (3.9%), computational neuroscience (3.9%), and machine learning (3.9%).

### fNIRS Datasets

The datasets analyzed by the groups for this study are publicly available at https://osf.io/b4wck^74^. The data are stored in SNIRF format^21^ and organized according to BIDS compliance^75^. The SNIRF files were validated using pysnirf2 version 0.7.4.

Dataset I (auditory tasks) contains fNIRS data from 17 healthy adults (age: 22-40 years) with no history of auditory disorders. Data were collected under the Macquarie University Ethics Committee and are published elsewhere^76^. The experimental paradigm consisted of three conditions: speech, noise, and silence (control). The speech stimulus consisted of three concatenated sentences with a total duration of 5.25 s. The noise stimulus consisted of a uniform distribution of frequency content between 300 and 700 Hz and was of 5 s duration. The control condition was a 5 s duration of silence. Stimuli were presented in a random order with an inter-stimulus interval ranging between 10 and 20 s. Each condition was presented 20 times.

The fNIRS probe for Dataset I contained 12 sources and 12 detectors resulting in 24 source-detector long-separation channels and 8 short-separation channels, and covered the left inferior frontal gyrus (IFG), the left and right superior temporal gyri (STG) and the occipital lobe. Data were acquired using a continuous-wave fNIRS device with source wavelengths of 760 and 850 nm (NIRScout, NIRx Technologies, Germany) and exported in native NIRx format. The data were then converted to BIDS format using the fNIRS-App sourcedata2bids using version 0.4.4^75^.

Dataset II (motor tasks) comprises fNIRS measurements from ten healthy, right-handed adults (age: 20-35 years) with no history of neurological or motor disorders. A subset of the dataset was previously published elsewhere^59^. In the experimental protocol, each subject completed four runs of a randomized block-designed protocol consisting of sequences of right- and left-hand finger tapping lasting 2 s and 3 s. The tasks were interleaved with rest periods ranging from 10 to 20 s, repeated 30 (2 s) and 25 (3 s) times. The protocol was approved by the University of Campinas Ethics Committee, where the experiment was carried out.

Data in Dataset II were acquired using a continuous-wave fNIRS device with source wavelengths of 760 and 850 nm (NIRScout, NIRx Technologies, Germany) at 8.9 Hz and exported in native NIRx format. For each participant, optodes positions were independently registered using an electromagnetic tracking device (Fastrak, Polhemus, USA), and these coordinates were manually integrated into the original data. The fNIRS probe contained 14 sources and 32 detectors covering the motor cortex, which allowed 64 source-detector pairs at approximately 3 cm and four pairs at 0.8 cm (short channels). After collection, the data were converted to BIDS format using MNE-BIDS version 0.10.

### Hypothesis Outcomes Reporting

We collected data from groups through two separate questionnaires: an Initial Survey and a Follow-up Questionnaire. The Initial Survey required groups to indicate for each hypothesis 1) whether it was confirmed (“yes”), refuted (“no”), or “not investigated”, 2) the outcome of any statistical tests (e.g., p-value, credible interval, or confidence intervals), if they were conducted, and 3) additional notes, along with a description of their analysis. The hypotheses given to the groups for the auditory and motor datasets are detailed in the Introduction section. Specifically for the motor dataset, groups had to submit individual reports for each of the ten participants.

The Follow-up Questionnaire was sent to all groups approximately six weeks after the Initial Survey’s deadline, requesting that they provide further details on the (1) programming language and/or analysis toolboxes used, (2) various steps of their analysis pipeline (from preprocessing to statistical analysis), and (3) their self-reported confidence both in their analysis skills and in the results submitted. For each analysis step mentioned in the questionnaire, groups could specify whether they employed the default settings of the analysis toolbox or, if not, the parameters used in their analysis.

### Analysis of Hypothesis Outcomes

The data collected from the questionnaires were analyzed by an independent group of researchers who had not submitted any reports themselves. This meta-analysis included a description of the demographics of the researchers, an examination of the responses to hypothesis testing and their association with the self-reported confidence levels, and an exploration of the different stages of the analysis pipelines and their effect on hypothesis testing outcomes. All analyses were carried out either in MATLAB (2023a, MathWorks, Natick, MA, USA) or in Python.

Sørensen-Dice similarity. The Sørensen-Dice similarity coefficient was used to assess the similarity of hypothesis results among groups. Pairwise similarity coefficients for each pair of vectors containing the results of all hypothesis tests were calculated as:

$${SD}\left({V}_{i},{V}_{j}\right)=2\,\times \frac{|{V}_{i}\cap {V}_{j}|}{|{V}_{i}|+|{V}_{j}|}$$

where ${{{{\boldsymbol{\ V}}}}}_{{{{\boldsymbol{\ i}}}}}, {{{{\boldsymbol{\ V}}}}}_{{{{\boldsymbol{\ j}}}}} \, {{{\in }}}\, \{{{{{\boldsymbol{\ V}}}}}_{{{{\mathbf{1}}}}},{{{{\boldsymbol{\ V}}}}}_{{{{\mathbf{2}}}}}, \ldots, {{{{\boldsymbol{\ V}}}}}_{{{{\boldsymbol{\ n}}}}}\}$ represent the responses of the *n* groups, $∣{{{\mathrm{V}}}_{{\mathrm{i}}}}∣$ and $|{{{\mathrm{V}}}_{{\mathrm{j}}}}|$ are the sizes of vectors ${{{\mathrm{V}}}_{{\mathrm{i}}}}$ and ${{{\mathrm{V}}}_{{\mathrm{j}}}}$, respectively, and $|{{{\mathrm{V}}}_{{\mathrm{i}}}}\cap{{{\mathrm{V}}}_{{\mathrm{j}}}}|$ denotes the cardinality of the intersection between ${{{\mathrm{V}}}_{{\mathrm{i}}}}$ and ${{{\mathrm{V}}}_{{\mathrm{j}}}}$. Sørensen-Dice calculations were performed in MATLAB.

Logistic regression. The relationship between the analysis steps (independent variables) and the hypothesis testing outcomes (dependent variable) across teams was investigated using binary logistic regression. The outcome of hypothesis testing was indicated by a binary variable, as reported by each team. As each independent variable includes multiple sub-categories (e.g., filtering can be divided into four categories - band-pass, high-pass, low-pass, and no filtering), we selected the most-commonly chosen category by the teams as the reference category. The logistic regression model was performed with the ‘fitglm’ function in MATLAB. Given the binary nature of the dependent variable, the binomial distribution was selected to appropriately model the response. The logistic link function, specifically the logit link, was applied to establish the connection between the linear predictor and the probability of observing a significant hypothesis testing outcome. This analysis was performed separately for each of the 47 hypotheses (7 at the group level and 40 at the individual level [i.e., 4 individual-level hypotheses x 10 subjects]).

We also employed multiple logistic regression to account for multiple independent variables, each representing a distinct step in the analysis. This method allowed us to assess the simultaneous impact of these analysis steps on the probability of obtaining a significant result in hypothesis testing. Prior to multiple logistic regression, we tested any multicollinearity between the processing pipeline steps that explain the variability in the hypothesis testing outcomes significantly (i.e., Pruning, HRF Estimation Method, and Signal Space) via a chi-squared test. This test is often applied to explore the association between categorical independent variables by identifying significant relationships between pairs of such variables. In this study, we conducted contingency table analysis to generate frequency distribution tables that illustrate the joint occurrences of categories for each pair of categorical independent variables. Subsequently, the chi-square test of independence was applied to evaluate the presence of significant associations between the categorical variables, which yielded both a test statistic and a *p*-value.

We established a *p*-value threshold of 0.05 for both the logistic regression analysis and the chi-square test.

Visualization of processing steps. We used Sankey diagrams (Python) to visualize essential processing steps and parameters the participating groups employed for signal analysis and statistical testing (Figs. 2, 3). The reported choice of methods, parameters, or regressors are encoded as nodes in the diagram. Each node belongs to one of several categories (for instance, a category of methods, such as “Artifact Rejection”), which make up the diagram’s vertical stages. This visualization enables both a quantitative and a qualitative assessment of the (co)occurrence and divergence of methodological choices across stages/categories in the analyses. It should be pointed out that the implied directionality of the stages is primarily a logical and not a temporal one and thus not strictly directional (for instance: “Motion Artifact Rejection” typically follows “Pruning”; the choice of a method for HRF estimation precedes the choice of a solver and the choice of regressors). The data were conditioned as follows before the construction of the Sankey diagrams: 1) Missing inputs, i.e., fields that were left empty and not reported otherwise by the researchers, were interpreted and categorized as a step not performed (e.g., no response in “Method used for artifact removal” implied no motion artifact removal performed). 2) Functionally equal or highly similar categories or nodes were joined (e.g., in “Quality Assessment and Pruning”, “Manual Selection” and “Visual Inspection of Time Domain” were joined into “Manual Pruning”). 3) In some categories, multiple choices and combinations of methods were reported. To account for this, we report the absolute frequencies of each method in % (i.e., the number of users that made that choice as a fraction of all users) and separately report numbers for both its individual use and its use in combination with others.

Groups reported using “default settings” for methods and parameters from different toolboxes and software packages used for analysis. We used an UpSet plot to report the frequency and intersections of categories for which the use of default settings was reported. The horizontal dimension visualizes the frequency for each individual category (e.g., about 47% of users applying default settings for “Filter Parameters”). The vertical dimension visualizes the intersection with other categories and how often these combinations were reported (e.g., 7 groups reported using default settings for all three categories “Filter Parameters”, “Artifact Correction Parameters,” and “Pruning Parameters”, but no other).

Association with self-reported confidence. Pearson’s correlation coefficient was employed to quantify the association between the self-reported confidence levels in analysis skills and the submitted results. We used the ‘corr’ function in MATLAB to compute the correlation matrix between the independent responses, from which we extracted Pearson’s correlation coefficient.

### Reporting summary

Further information on research design is available in the Nature Portfolio Reporting Summary linked to this article.

## Supplementary information

Reporting Summary

## Data Availability

The fNIRS datasets analyzed by the groups for this study are publicly available at https://osf.io/b4wck^74^. The metadata containing the anonymized reports analyzed in this work is publicly available as a .csv file (FreshData.csv) at https://github.com/ibs-lab/FRESH/tree/main/data.

## Code availability

All the Python and MATLAB scripts used to perform the analysis and to produce the figures and tables in this work are publicly available at https://github.com/ibs-lab/FRESH/.
