# Publisher Correction: fNIRS reproducibility varies with data quality, analysis pipelines, and researcher experience

**DOI:** 10.1038/s42003-025-08714-4

**Published:** 2025-08-21

**Authors:** Meryem A. Yücel, Robert Luke, Rickson C. Mesquita, Alexander von Lühmann, David M. A. Mehler, Michael Lührs, Jessica Gemignani, Androu Abdalmalak, Franziska Albrecht, Iara de Almeida Ivo, Christina Artemenko, Kira Ashton, Paweł Augustynowicz, Aahana Bajracharya, Elise Bannier, Beatrix Barth, Laurie Bayet, Jacqueline Behrendt, Hadi Borj Khani, Lenaic Borot, Jordan A. Borrell, Sabrina Brigadoi, Kolby Brink, Chiara Bulgarelli, Emmanuel Caruyer, Hsin-Chin Chen, Christopher Copeland, Isabelle Corouge, Simone Cutini, Renata Di Lorenzo, Thomas Dresler, Adam T. Eggebrecht, Ann-Christine Ehlis, Sinem B. Erdoğan, Danielle Evenblij, Talukdar Raian Ferdous, Victoria Fracalossi, Erika Franzén, Anne Gallagher, Christian Gerloff, Judit Gervain, Noy Goldhamer, Louisa K. Gossé, Ségolène M. R. Guérin, Edgar Guevara, SM Hadi Hosseini, Hamish Innes-Brown, Isabell Int-Veen, Sagi Jaffe-Dax, Nolwenn Jégou, Hiroshi Kawaguchi, Caroline Kelsey, Michaela Kent, Roman Kessler, Nadeen Kherbawy, Franziska Klein, Nofar Kochavi, Matthew Kolisnyk, Yogev Koren, Agnes Kroczek, Alexander Kvist, Chen-Hao Paul Lin, Andreas Löw, Siying Luan, Darren Mao, Giovani G. Martins, Eike Middell, Samuel Montero-Hernandez, Murat Can Mutlu, Sergio L. Novi, Natacha Paquette, Ishara Paranawithana, Yisrael Parmet, Jonathan E. Peelle, Ke Peng, Tommy Peng, João Pereira, Paola Pinti, Luca Pollonini, Ali Rahimpour Jounghani, Vanessa Reindl, Wiebke Ringels, Betti Schopp, Alina Schulte, Martin Schulte-Rüther, Ari Segel, Tirdad Seifi Ala, Maureen J. Shader, Hadas Shavit, Arefeh Sherafati, Mojtaba Soltanlou, Bettina Sorger, Emma Speh, Kevin D. Stubbs, Katharina Stute, Eileen F. Sullivan, Sungho Tak, Zeus Tipado, Julie Tremblay, Homa Vahidi, Maaike Van Eeckhoutte, Phetsamone Vannasing, Gregoire Vergotte, Marion A. Vincent, Eileen Weiss, Dalin Yang, Gülnaz Yükselen, Dariusz Zapała, Vit Zemanek

**Affiliations:** 1 Boston University, Neurophotonics Center, Boston, MA USA; 2 Department of Biomedical Engineering, College of Engineering, Boston University, Boston, MA USA; 3 Department of Linguistics, Macquarie University, Sydney, NSW Australia; 4 Australia Hearing Hub, Macquarie University Hearing, Sydney, NSW Australia; 5 School of Computer Science, University of Birmingham, Birmingham, UK; 6 Institute of Physics, University of Campinas (UNICAMP), Campinas, São Paulo, Brazil; 7 Intelligent Biomedical Sensing (IBS) Lab, Machine Learning Department, TU Berlin, Berlin, Germany; 8 BIFOLD – Berlin Institute for Foundations of Learning and Data, Berlin, Germany; 9 Applied Computational Neuroscience (ACN) lab, Department for Psychiatry, Psychotherapy and Psychosomatics, Medical School, RWTH Aachen University, Aachen, Germany; 10 Institute of Translational Psychiatry, Medical Faculty, University of Münster, Münster, Germany; 11 School of Psychology, Cardiff University Brain Research Imaging Center (CUBRIC), Cardiff University, Cardiff, UK; 12 Department of Cognitive Neuroscience, Maastricht University, Maastricht, The Netherlands; 13 Department of Developmental Psychology and Socialization, Università degli Studi di Padova, Padua, Italy; 14 Padova Neuroscience Center, Padua, Italy; 15 Brain and Mind Institute, Western University, London, ON Canada; 16 Department of Physiology and Pharmacology, Western University, London, ON Canada; 17 Division of Physiotherapy, Department of Neurobiology, Care Sciences and Society, Karolinska Institutet, Stockholm, Sweden; 18 Karolinska University Hospital, Women’s Health and Allied Health Professionals Theme, Medical Unit Occupational Therapy, Stockholm, Sweden; 19 Department of Psychology, University of Tübingen, Tübingen, Germany; 20 Department of Neuroscience, American University, Washington, WA USA; 21 Department of Experimental Psychology, The John Paul II Catholic University of Lublin, Lublin, Poland; 22 Mallinckrodt Institute of Radiology, Washington University School of Medicine, St. Louis, WA USA; 23 CHU Rennes, Radiology Department, Rennes, France; 24 Univ Rennes, Inria, CNRS, Inserm, IRISA UMR 6074, Empenn, Rennes, France; 25 Department of Psychiatry and Psychotherapy, Tübingen Center for Mental Health, University of Tübingen, Tübingen, Germany; 26 LEAD Graduate School & Research Network, University of Tübingen, Tübingen, Germany; 27 German Center for Mental Health (DZPG), partner site, Tübingen, Germany; 28 BEARLabs, Faculty 1, University of Applied Sciences (HTW) Berlin, Berlin, Germany; 29 Research Institute for Sport and Exercise Sciences, Liverpool John Moores University, Liverpool, England; 30 Department of Biomechanics, University of Nebraska at Omaha, Omaha, NE USA; 31 Department of Occupational Therapy Education, University of Kansas Medical Center, Kansas City, KS USA; 32 Centre for Brain and Cognitive Development, Birkbeck, University of London, London, UK; 33 Department of Psychology, National Chung Cheng University, Minhsiung, Chiayi, Taiwan; 34 Division of Developmental Medicine, Department of Pediatrics, Boston Children’s Hospital, Boston, MA USA; 35 Harvard Medical School, Boston, MA USA; 36 Faculty of Engineering and Natural Sciences, Department of Biomedical Engineering, Acıbadem Mehmet Ali Aydınlar University, Istanbul, Turkey; 37 Department of Biomedical Engineering, University of Houston, Houston, TX USA; 38 Department of Psychology, University of Montreal, Montreal, QC Canada; 39 Department of Child and Adolescent Psychiatry, RWTH Aachen University, Aachen, Germany; 40 JARA-Brain Institute II, Molecular Neuroscience and Neuroimaging, RWTH Aachen & Research Centre Juelich, Aachen, Germany; 41 The Translational Neurorehabilitation Lab at Adi Negev Nahalat Eran, Ofakim, Israel; 42 Institute of Neuroscience, Université Catholique de Louvain, Brussels, Belgium; 43 Sciences Cognitives et Sciences Affectives, University of Lille, CNRS, Lille, France; 44 CIACYT, Universidad Autónoma de San Luis Potosí, San Luis Potosí, Mexico; 45 Department of Psychiatry and Behavioral Sciences, Computational Brain Research and Intervention (C-BRAIN) Lab, Stanford University, Stanford, CA USA; 46 Eriksholm Research Centre, Snekkersten, Denmark; 47 Department of Health Technology, Technical University of Denmark, Lyngby, Denmark; 48 School of Psychological Sciences and Sagol School of Neuroscience, Tel Aviv University, Tel Aviv, Israel; 49 Human Informatics and Interaction Research Institute, National Institute of Advanced Industrial Science and Technology (AIST), Tsukuba, Japan; 50 Schulich School of Medicine and Dentistry, Western University, London, ON Canada; 51 Department of Neuropsychology, Max Planck Institute for Human Cognitive and Brain Sciences, Leipzig, Germany; 52 Neurocognition and functional Neurorehabilitation Group, Department of Psychology, University of Oldenburg, Oldenburg, Germany; 53 Biomedical Devices and Systems Group, R&D Division Health, OFFIS Institute for Information Technology, Oldenburg, Germany; 54 Department of Psychology, Western University, London, ON Canada; 55 Department of Physical Therapy, Ben-Gurion University, Beersheba, Israel; 56 Department of Physics, Washington University in St. Louis, St. Louis, MO USA; 57 Experimental Psychology Unit, Helmut Schmidt University/University of the Federal Armed Forces Hamburg, Hamburg, Germany; 58 Health and Rehabilitation Sciences Program, Faculty of Health Science, Western University, London, ON Canada; 59 Human Hearing, The Bionics Institute, East Melbourne, VIC Australia; 60 Institute of Biology, Otto von Guericke University, Magdeburg, Germany; 61 Industrial engineering and management, Ben-Gurion university, Beersheba, Israel; 62 Department of Communication Sciences and Disorders and Department of Psychology, Northeastern University, Boston, MA USA; 63 Department of Electrical and Computer Engineering, Price Faculty of Engineering, University of Manitoba, Winnipeg, MB Canada; 64 Coimbra Institute for Biomedical Imaging and Translational Research, Institute for Nuclear Sciences Applied to Health – University of Coimbra, Coimbra, Portugal; 65 Department of Engineering Technology, University of Houston, Houston, TX USA; 66 Department of Psychology, University of California, Merced, Merced, CA USA; 67 Psychology, School of Social Sciences, Nanyang Technological University, Singapore, Republic of Singapore; 68 Department of Experimental Otology of the Clinics of Otolaryngology, Hannover Medical School, Hannover, Germany; 69 Department of Child and Adolescent Psychiatry and Psychotherapy, University Medical Center Göttingen, Göttingen, Germany; 70 Department of Child and Adolescent Psychiatry and Psychotherapy, University Hospital Heidelberg, Heidelberg, Germany; 71 Department of Speech, Language, and Hearing Sciences, Purdue University, West Lafayette, IN USA; 72 Department of Neurology, University of California, San Francisco, CA USA; 73 School of Psychology, University of Surrey, Guildford, UK; 74 Department of Childhood Education, Faculty of Education, University of Johannesburg, Johannesburg, South Africa; 75 BrainsCAN, Western University, London, ON Canada; 76 Chemnitz University of Technology, Faculty of Behavioural and Social Sciences, Department of Human Movement Science and Health, Chemnitz, Germany; 77 Center for Bio-Imaging and Translational Research, Korea Basic Science Institute, Cheongju, Republic of Korea; 78 Graduate School of Analytical Science and Technology, Chungnam National University, Daejeon, Republic of Korea; 79 Department of Psychopharmacology, Maastricht University, Maastricht, The Netherlands; 80 Copenhagen Hearing and Balance Centre, Rigshospitalet, Copenhagen, Denmark; 81 EuroMov Digital Health in Motion, Univ Montpellier, IMT Mines Ales, Montpellier, France; 82 Department of Clinical Neuropsychology, University Hospital RWTH Aachen, Aachen, Germany; 83 Faculty of Science, University of Amsterdam, Amsterdam, The Netherlands

**Keywords:** Neuroscience, Research data

Correction to: *Communications Biology* 10.1038/s42003-025-08412-1, published online 4 August 2025

In this article, Meryem A. Yücel should have been denoted as a co-corresponding author. The original article has been corrected.

